# Publisher's Note

**DOI:** 10.1080/20008198.2018.1540192

**Published:** 2018-11-02

The following Letter to the Editor was originally published in issue 9(1). It has now been added to issue 8(S6) (Special Issue: Traumatic loss):

Response to: Prolonged grief disorder for ICD-11: The primacy of clinical utility and international applicability

Maarten C. Eisma^a^ and Lonneke I. M. Lenferink^a^^,^^b^

^a^Department of Clinical Psychology and Experimental Psychopathology, Faculty of Behavioral and Social Sciences, University of Groningen, Groningen, The Netherlands; ^b^Department of Clinical Psychology, Faculty of Social Sciences, Utrecht University, Utrecht, The Netherlands

DOI: 10.1080/20008198.2018.1512249

Special Issue URL: https://tandfonline.com/toc/zept20/8/sup6?nav=tocList

